# Diagnosis

**Published:** 1874-01

**Authors:** E. S. Chisholm


### DENTAL REGISTER.

VOL. XXVIII.] JANUARY, 1874. [No. 1.

### DIAGNOSIS.

BY E. S. CHISHOLM.

That branch of medicine accurately predicated upon a knowledge of disease, which suggests the necessary curative indications for such disease, is significantly styled *Diagnosis*.

This word, according to Dr. Harris, implies literally, "I know;" and its medical definition implies the idea of power or means of discriminating disease, and marking the distinguishing characteristics of one disease from another.

This branch of the healing art is dependent upon pathological conditions, being properly the offspring of disease, or the summary of a well grounded knowledge of it. And as the knowledge of the practitioner of medicine or dentistry increases, commensurate with it does his power of discrimination extend.

A proper standard of this branch of medical education can only be attained by a correct understanding of tissue, both in its normal and abnormal state ; since it is by contrast of organs in their known physiological condition with those in a diseased state that we are able to make a correct diagnosis—recognizing and appreciating their changes, extent, locality and relative influences.

As before stated, diagnosis is predicated upon an understanding of pathology ; likewise is the treatment of disease founded on diagnosis. Hence, the sum of a disease must be accurately understood before the proper remedial agents can be applied with any certainty of cure.

There have been, more or less, in all ages, two systems or schools of medical treatment : of which the one prevails among the ignorant or more uncultivated,in rude states of society, and is known as the empirical ; the other requiring a higher degree of enlightenment, and is termed the rational system. The first is an inductory system, in which by accident or otherwise a certain kind of medicine is found to be good in the treatment of a disorder, and on a number of such data, a system is predicated. The rational system is founded on a higher order of knowledge ; as for instance when many thousands of cases are compared, and inferences drawn from these comparisons. This system of course was empirical in its primitive day, but through successive ages have investigations and developments been made, step by step, and recorded in and transferred through the medium of books, until it has received the comeliness of an extensive science. Therefore, for the proper perfection of medicine as a rational system, or science, two things are indispensable ; first, a right understanding of causes and symptoms of disease : and second, a right knowledge of the action of medicine. When these two subjects are understood, all will have been attained that medicine will promise. The first of these—a right knowledge of *causes* and *symptoms*—is the subject now under consideration. Developments are rapidly being made in this department of medical science ; and greatly outrivaling the other end of the rational system—or that of a correct knowledge of the action of medicine. So we will likely know, soon, what we have to cure, if we do not know the remedy

All diseases have their origin in causes producing certain pernicious effects; yet it does not follow that should such causes be removed, the effects will also, than that, should the acorn from which the stately oak sprung be removed, the oak would also. To prick the flesh with a thorn may produce fatal tetanus, though the thorn be removed. In many instances, however, the assistance of nature after exciting causes are withdrawn will be all that is necessary to effect a cure of many maladies, depending on the amount of disorganization induced, extent of lesions, duration of contact, constitutional and predisposing tendencies.

Causes frequently produce effects, that are themselves soon inaugurated causes of still greater malignant influence, thus extending step by step until the whole system becomes involved, and death supervenes. The animal body, therefore, is an organized unit wherein one organ is dependent upon another for support; and where, one organ laboring under the weight of disease, the whole organism may suffer therefrom, even though in very remote regions. The harmonious action of a piece of machinery may be disturbed by interposing an insignificant foreign substance; also may our physical organization; and that, too, from very trivial causes be so much deranged as to entirely arrest the functional movements of the whole.

It is not uncommon for the medical practitioner, much more frequently the dentist, to look at immediate or exciting causes, with no regard whatever for predisposing and constitutional tendencies. Diagnosis thus predicated is strictly building upon a sandy foundation, and only directs to lôcal treatment. When, however, local irritation is prevalent, the intensity is greatly increased by tendencies. We would instance when a small deposit of tartar on the neck of a lower tooth would cause the gum to assume a dark livid color and produce a thickening of the gum, and bleed at the slightest touch and would be far less susceptible to therapeutic agents, while the same condition in a subject free from such tendencies would only produce a slight marginal redness of the gum and requiring no treatment other than its removal.

In symptomatology we are not to disregard the statements of our patients. After some years' experience with human nature, it is no difficult task to read an expression of fear or timidity on the countenance and be better prepared for their statements which will certainly have an inclination towards their preferences or feelings.

It were better, however, for the less experienced practitioner to receive their statements with caution and *never* direct a treatment or operation upon our patient's judgment. Through fear of our promptly deciding upon a painful operation they often evade our interrogations and will not acknowledge the intensity nor duration of pain they have undergone. Others may mislead us by failing to understand our expressions of pain, etc.; for instance do, not appreciate the difference between sensitive points on the teeth or pain from absolute pulp exposure. Children may say their "*teeth are aching*" when we are excavating, for a want of understanding of words. An explanation is here necessary before they can understand us, or we them. Contact with a foreign substance produces sensation only; while in the other, the substance may be removed and the pain lingers. The sensations are very different and can easily be so explained as that a child may comprehend the difference.

Sometimes patients feign a greater suffering than they really experience under our operating. For this a test may be made with chloride of zinc, unless contra-indicated, when its application will occasion a little or no pain and proportionate with the intensity will sensitive dentine be obtunded.

As to locality, complaint is often made of pain very remote from the affecting cause; as for instance, the tiring pain felt in the articular region of the lower jaw from a diseased lower molar or perhaps a dens sapientiæ. Not unfrequent is it from sensations most distressing to be felt in one jaw when really the exciting cause is in the other, or the other side of the mouth. These facts prove the useless trusting of our patients' statements of their own maladies.

Percussion is equally valuable in dentistry as in physical diagnosis. By the simple tapping of the teeth consecutively, of the diseased side, a response of soreness will be felt when the affected tooth is reached. If pain has supervened for several days, more than one tooth will be likely be sore from sympathy, yet the exciting cause will be the most intense. Sometimes where affections are slight only a lateral pressure in a vibrating manner with the thumb and finger will be necessary to reveal the locality.

I have found this test made with the handle of an instrument where there exists any abnormal tendencies whatever, either nervous or periosteal, acute or chronic, most generally correct.

The more thoroughly developed branch of medicine teaches the use of numerous invented appliances for inspecting disease, and not only through aid to sight, but also touch, hearing and smell. Thermometers reveal temperatures of various parts of the body, specific-gravity bottles and other measures give the relative gravity of fluids, the stethescope bespeaks the condition of the chest and organs of breathing. With the dental surgeon, however, his mouth, mirror and delicate pointed instruments are indispensable requisites in detecting normal and abnormal conditions of the teeth. The mirror is strictly a diagnostic agent, while the excavator may be a therapeutic implement as well. "*Useless each without the other.*"

The localities of diseases of the teeth, if perceptible at all externally, are revealed by these agents, when the proper therapeutic indications will be manifest. And we deem it not digressing too far here to remark that we cannot be too cautious in examining the teeth. For with the confidence our patients impose in us and pay us besides, honesty would direct our closest scrutiny before pronouncing upon them. It would be empirical to falsely impress our patients with regard to the condition of their teeth either for remunerative means or want of skill. Honesty and candor is the best policy. Every visible surface of the teeth should be closely scanned in a systematic way ; and when they are in contact so close as to prevent close inspection, separation should be made with a file or wedge before a diagnosis is made.

Having found few only in the dental profession who make use of their olfactory senses in diagnosis, I will take occasion here to remark that the nose has much to do with nosology

Where purulent discharges exist in carious teeth, with either exposed or devitalized pulps, to probe with a broach and pass in front of the nose speaks in loud terms the contraindication to filling without first restoring the organ to health.

The late Dr. C. A. Harris made mention of this fact many years ago to his students, but, not having seen a mention of it in any writings of his, I deem it not out of place to recommend it as a diagnostic indication. The same fact may be arrived at by questioning our patients as to offensive discharges ; yet if such exist they will probably fail to know the locality or to have even noticed them.

During each operation, we should be cautious to avoid regular beaten paths that would cause our neglect to observe any new symptoms than might be presented. For instance, should the caries in a tooth so nearly expose the pulp that a metallic filling would cause pain from pressure or thermal change, a deviation would be indicated. Again, should two fillings differently electrified be in contact, a galvanic current would result and pain be produced.

The dental profession, though making rapid strides to a higher standard of excellence, is yet sadly deficient in a thorough knowledge of the pathology and symptomatology of the teeth. All efforts to the present time merely give us stand-points from which to view the expansive and unexplored region in our front. The delicacy of tissue with which we have to deal, the complication of nervous ramification, the sympathetic relation of the teeth with other parts, all contribute to obscure the pathological condition and render diagnosis difficult. Yet, with all these difficulties, we are gradually ascending to the goal of excellence, and though we may not know how to cure, we will probably know what is to cure.

Chemistry with its most searching analyses, and the microscope with its revealed wonders, also the daily habit of investigation,—each directing how to classify the diseased signs they expose in connection with each other and considering respectively their differential bearing,—may yet with other auxiliaries aid in developing a well-grounded system worthy of our boasting.

It is useless for me in a short essay to submit to your consideration more than a few general points, since the subject is too vast. It would take an immense volume to give the symptomatology of all the diseases of the face and head or wherein the dental practitioner's skill is called in; our object here having been only to write *about*, rather than *on* this subject of diagnosis. Age of patients, habits, occupation, condition, whether any known or hereditary cause, besides the necessary examination to local or exciting causes, shall all be balanced and duly considered.

The condition of the weather, too, must not be overlooked. The human system is a good barometer and expresses with much accuracy the changes of the weather.

The observant practitioner well knows its influence upon the aged, chronic diseases, old sores, gunshot wounds, rheumatism, neuralgia, etc. Before a rain the system is more vascular, secretions more profuse, with feelings of languor, and much more susceptible to catarrhal affections.

Who among you have not noticed the increased calls on account of aching teeth, and how unyielding to treatment when the atmosphere is warm and humid, with lowering clouds and indications of rain! This statement, though it may be regarded irrelevant, since we cannot change the weather, yet, in looking to causes and symptoms, may induce us to use only palliatives to relieve immediate pain, and defer all operations when inflammation might be superinduced, until favorable weather.

